# The Molecular Architecture of Neurodegeneration: An Integrative Overview of Convergent Mechanisms

**DOI:** 10.3390/neurosci7010007

**Published:** 2026-01-06

**Authors:** Gonzalo Emiliano Aranda-Abreu, Fausto Rojas-Durán, María Elena Hernández-Aguilar, Deissy Herrera-Covarrubias, Luis Roberto Tlapa-Monge, Sonia Lilia Mestizo-Gutiérrez

**Affiliations:** 1 Instituto de Investigaciones Cerebrales, Universidad Veracruzana, Xalapa 91190, Veracruz, Mexico; frojas@uv.mx (F.R.-D.); elenahernandez@uv.mx (M.E.H.-A.); dherrera@uv.mx (D.H.-C.); 2 Doctorado en Investigaciones Cerebrales, Universidad Veracruzana, Xalapa 91190, Veracruz, Mexico; roberto.tlapa@outlook.com; 3 Facultad de Ciencias Químicas, Universidad Veracruzana, Xalapa 91000, Veracruz, Mexico; smestizo@uv.mx

**Keywords:** neurodegeneration, oxidative stress, proteostasis, neuroinflammation, therapeutic strategies

## Abstract

Neurodegenerative diseases such as Alzheimer’s, Parkinson’s, amyotrophic lateral sclerosis, and Huntington’s disease represent a major challenge in neuroscience due to their complex, multifactorial nature and the absence of curative treatments. These disorders share common molecular mechanisms, including oxidative stress, mitochondrial dysfunction, proteostasis collapse, calcium dyshomeostasis, chronic neuroinflammation, and the prion-like propagation of misfolded proteins. Together, these processes trigger a cascade of cellular damage that culminates in synaptic dysfunction and programmed neuronal death. This review integrates current evidence on the sequential stages of neurodegeneration, emphasizing the convergence of oxidative, inflammatory, and proteotoxic pathways that drive neuronal vulnerability. Moreover, it explores emerging therapeutic strategies aimed at restoring cellular homeostasis, such as Nrf2 activation, modulation of the unfolded protein response (UPR), enhancement of autophagy, immunotherapy against pathological proteins, and gene therapy approaches. The dynamic interplay among mitochondria, endoplasmic reticulum, and glial cells is highlighted as a central element in disease progression. Understanding these interconnected mechanisms provides a foundation for developing multi-targeted interventions capable of halting or delaying neuronal loss and improving clinical outcomes in neurodegenerative disorders. This work provides an integrative and introductory overview of the convergent mechanisms underlying neurodegeneration rather than an exhaustive mechanistic analysis.

## 1. Introduction

One of the main challenges in neuroscience is neurodegenerative diseases, which to this day remain incurable and represent a major public health issue.

The increase in life expectancy has led to a higher prevalence of CNS disorders, whose clinical manifestations center on cognitive decline, motor impairments, sensory dysfunction, and behavioral changes. These symptoms reflect a progressive and irreversible loss of neuronal circuits [1].

Understanding the convergent mechanisms that drive neurodegeneration is of critical importance due to the growing global impact of AD, PD, ALS, and HD. Together, these disorders represent one of the leading causes of disability and death worldwide, with increasing prevalence driven by demographic aging. Despite their distinct etiologies, they share molecular pathways—such as mitochondrial dysfunction, proteostasis collapse, chronic neuroinflammation, and prion-like protein propagation—that collectively determine neuronal vulnerability and disease progression. Integrating these mechanisms into a unified framework is essential not only for improving early diagnosis and biomarker discovery but also for guiding the development of multi-target therapeutic strategies capable of addressing the complex and interconnected nature of neurodegeneration. This broader perspective highlights the urgent need for mechanistic synthesis to inform translational research and future interventions.

Although each pathology has a different etiology, there is a convergence of pathogenic mechanisms, suggesting the existence of common cellular routes leading to neurodegeneration [2]. From a molecular perspective, neurodegeneration can be understood as an imbalance between cellular defense mechanisms and processes that induce structural and metabolic damage. The former include antioxidant capacity, proteasome- and autophagy-mediated protein degradation, and the homeostatic function of glial cells. The latter include oxidative stress, mitochondrial dysfunction, endoplasmic reticulum alterations, excitotoxicity, and chronic inflammation [3].

From a clinical perspective, various symptoms reflect the vulnerability of different neuronal types. In AD, the accumulation of β-amyloid peptide and hyperphosphorylated Tau protein causes synaptic loss and cognitive decline [4]. In PD, the degeneration of dopaminergic neurons in the substantia nigra pars compacta is associated with the accumulation of α-synuclein in Lewy bodies [5]. In ALS, TDP-43 disorganization and excitotoxicity mediate the loss of spinal motor neurons [6], while in HD, CAG triplet expansion in the HTT gene leads to a protein prone to aggregation and progressive neurotoxicity [7].

These pathologies converge in the neurons’ inability to adapt to chronic stress conditions, leading to synaptic dysfunction and, ultimately, cell death. The accumulation of misfolded proteins, mitochondrial dysfunction, and persistent glial inflammation creates an environment where repair mechanisms become insufficient.

Therefore, neurodegeneration is a self-sustaining process in which initial neuronal death triggers cellular responses that amplify the damage, affecting previously intact circuits [8].

The study of these mechanisms allows us to reformulate the concept of neurodegeneration—from a systemic to an integrative approach. Given the breadth of the topic, this review is intended as a conceptual introductory synthesis aimed at students and early-stage researchers, rather than an in-depth mechanistic analysis of each pathway. Understanding these mechanisms enables the design of comprehensive therapies aimed at restoring neuronal homeostasis and halting disease progression.

**Figure 1:**
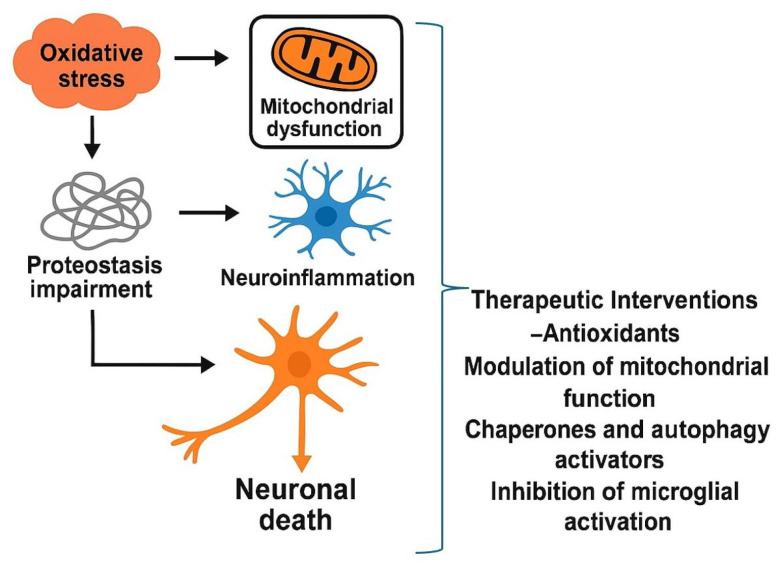
Schematic representation of interconnected pathogenic pathways leading to neuronal death. Oxidative stress and mitochondrial dysfunction contribute to proteostasis impairment and chronic neuroinflammation, which together drive progressive neuronal loss. Potential therapeutic interventions include antioxidants, modulation of mitochondrial function, activation of chaperones and autophagy, and inhibition of microglial activation.

The objective of this review is to describe the stages leading to a neurodegenerative process, focusing on AD, PD, ALS, and HD (Figure 1).

## 2. Methodology

This narrative review was conducted using a structured literature search across PubMed, Scopus, and Web of Science from 1990 to 2025. The following keywords and Boolean combinations were used: “neurodegeneration”, “oxidative stress”, “mitochondrial dysfunction”, “proteostasis”, “UPR”, “ER stress”, “prion-like propagation”, “neuroinflammation”, and “synaptic dysfunction”, combined with terms such as “Alzheimer’s disease”, “Parkinson’s disease”, “ALS”, and “Huntington’s disease”. Peer-reviewed original articles, reviews, and meta-analyses published in English were included. Studies were excluded if they were non-neurological proteinopathies, non-peer-reviewed sources, or articles lacking mechanistic relevance. Additional references were selected through backward citation tracking to ensure comprehensive coverage of key mechanistic pathways. This methodological approach ensures that the review integrates the most relevant and up-to-date evidence across molecular, cellular, and translational domains.

## 3. Oxidative Stress and Mitochondrial Dysfunction

Oxidative stress constitutes one of the earliest events in the neurodegenerative cascade. It is defined as an imbalance between the generation of reactive oxygen species (ROS) and reactive nitrogen species (RNS) and the cell’s antioxidant capacity to neutralize them [9]. In the CNS, this process acquires particular relevance due to the high metabolic demand of neurons, their high lipid content, and the relative scarcity of antioxidant systems, which makes them vulnerable to oxidation [10]. The accumulation of free radicals damages lipids, proteins, and DNA, leading to progressive dysfunction that affects neuronal and synaptic integrity.

### 3.1. Reactive Species and Neuronal Vulnerability

Mitochondria represent the main source of ROS. During oxidative phosphorylation, electron flow through complexes I and III of the respiratory chain generates superoxide (O_2_^−^), which can be converted into hydrogen peroxide (H_2_O_2_) and hydroxyl radicals (•OH). Under physiological conditions, these reactive species are neutralized by antioxidants such as superoxide dismutase (SOD), catalase, and glutathione peroxidase (GPx). However, when ROS production exceeds neutralization capacity, structural alterations occur in mitochondrial proteins, mtDNA, and membrane lipids [11].

Neurons are particularly vulnerable to this type of damage due to their high metabolic rate and limited regenerative capacity. Moreover, the highly polarized structure of neurons—where the soma and synapses are separated by long axons—requires continuous mitochondrial transport to distal regions. When this transport is compromised, mitochondria cannot reach synapses efficiently, reducing local ATP availability and increasing vulnerability to stress. Examples include the proteins Miro and Milton, which accumulate with dysfunctional organelles in axons and dendrites, contributing to distal degeneration [12,13,14].

### 3.2. Mitochondrial Dysfunction and Energy Damage

Mitochondrial dysfunction not only increases ROS generation but also impairs ATP production, resulting in energy deficits and oxidative stress. The loss of mitochondrial membrane potential (ΔΨm) disrupts calcium homeostasis and ATP synthesis, directly affecting neurotransmitter release and synaptic plasticity.

Studies on PD have shown that inhibition of complex I by neurotoxins such as MPTP(1-methyl-4-phenyl-1,2,3,6-tetrahydropyridine), a compound widely used to model PD, acts as a pro-toxin. After crossing the blood–brain barrier, it is metabolized by astrocytic monoamine oxidase-B (MAO-B) into MPP^+^ (1-methyl-4-phenylpyridinium), the active neurotoxic species that selectively impairs mitochondrial complex I in dopaminergic neurons. reproduces the dopaminergic degeneration observed in patients, demonstrating the central role of mitochondria in pathogenesis [15,16,17].

In AD, decreased activity of complexes III and IV, along with increased mitochondrial fragmentation mediated by Drp1, promotes cytochrome c release and the activation of pro-apoptotic caspases [18].

In ALS, mutations in SOD1 impair superoxide detoxification and promote the formation of protein aggregates that damage mitochondrial membranes [19]. In HD, the mutant huntingtin protein interferes with the function of mitochondrial permeability transition pore proteins, triggering apoptosis [20,21].

### 3.3. Antioxidant Defense Mechanisms and Selective Vulnerability

The brain possesses several endogenous antioxidant systems, both enzymatic and non-enzymatic. The enzymatic systems include SOD isoforms, catalase, GPx, and peroxiredoxins, while the non-enzymatic ones comprise reduced glutathione (GSH), ascorbic acid, and vitamin E [22]. Under normal conditions, the transcription factor Nrf2 (nuclear factor erythroid 2-related factor 2) regulates the expression of antioxidant genes upon ROS increase [23]. However, in neurodegenerative diseases, dysfunction of this pathway has been described, limiting the nervous system’s ability to adequately respond to oxidative stress [24].

The selective vulnerability of certain neuronal populations—such as dopaminergic neurons in Parkinson’s disease or motor neurons in ALS—can be partially explained by intrinsic metabolic features and differential exposure to toxic stimuli. For example, dopaminergic neurons are especially sensitive to mitochondrial toxins such as MPP^+^ (the active metabolite of MPTP) and pesticides like rotenone, both of which selectively inhibit mitochondrial complex I, a process to which these neurons are highly susceptible due to their elevated oxidative burden and pacemaking activity. In ALS, motor neurons are preferentially affected by excitotoxic levels of glutamate, resulting from impaired astrocytic uptake, which leads to excessive Ca^2+^ influx and downstream mitochondrial stress. These specific neurotoxic mechanisms, combined with differences in mitochondrial density, oxidative load, and calcium handling, contribute to the selective vulnerability observed across neurodegenerative diseases [25,26].

### 3.4. Interaction Between Oxidative Stress and Inflammation

Oxidative stress and inflammation reinforce each other in a process known as oxidative inflammation. Microglial activation produces nitric oxide (NO) through iNOS, which reacts with superoxide to form peroxynitrite (ONOO^−^), one of the most damaging molecules to neuronal proteins. This process, described in AD and PD, promotes the nitration of structural and enzymatic proteins, impairing their function [27,28]. ROS can activate proinflammatory transcription factors such as NF-κB, increasing the release of cytokines like TNF-α and IL-1β, which amplify neuronal damage [29,30].

### 3.5. Long-Term Consequences

Persistent oxidative stress induces genomic, epigenetic, and proteomic alterations. In particular, mitochondrial DNA damage produces cumulative mutations that aggravate energy impairment. At the epigenetic level, oxidation of histones and DNA can modify gene expression, affecting pathways related to cell survival and neuroplasticity [31,32].

Numerous in vivo and in vitro studies support the central role of oxidative stress and mitochondrial dysfunction in neurodegeneration. MPTP-treated mouse models reproduce dopaminergic mitochondrial failure characteristic of PD [33,34], while SOD1^G93A transgenic mice demonstrate mitochondrial swelling, impaired ATP production, and increased ROS generation consistent with ALS pathology [35,36]. Similarly, primary neuronal cultures exposed to Aβ oligomers show reduced mitochondrial membrane potential and elevated oxidative markers [37]. These experimental findings support the mechanistic framework presented in this section.

## 4. Response to Protein Misfolding and Endoplasmic Reticulum Stress

The accumulation of misfolded proteins is a hallmark of neurodegenerative diseases. Under physiological conditions, neurons maintain a proteostatic balance that ensures proteins reach their correct tertiary or quaternary structures, while those that are misfolded or damaged are promptly degraded. Proteostasis involves the coordinated action of the endoplasmic reticulum (ER), the ubiquitin–proteasome system, and lysosomal autophagy [38,39].

When the capacity of these systems is overwhelmed by an excess of misfolded proteins, an adaptive response known as the unfolded protein response (UPR) is activated. Its initial goal is to restore homeostasis; however, if stress persists, the UPR can induce apoptosis and contribute to neuronal degeneration [40].

### 4.1. The ER as a Sensor of Proteotoxic Stress

The ER plays a central role in protein synthesis, folding, and post-translational modification. Genetic mutations, oxidative stress, mitochondrial dysfunction, or calcium imbalance can impair its function, leading to the accumulation of misfolded or improperly assembled proteins in the lumen. This stressful state activates three transmembrane sensors: PERK (protein kinase RNA-like ER kinase), IRE1α (inositol-requiring enzyme 1 alpha), and ATF6 (activating transcription factor 6) [41].

PERK is a kinase that phosphorylates the factor eIF2α, globally reducing protein synthesis while allowing the selective translation of ATF4, which regulates antioxidant and pro-apoptotic genes such as CHOP [42].

IRE1α functions as an endoribonuclease that cleaves XBP1 mRNA, generating an active form (XBP1s) that induces molecular chaperones and components of the ER-associated degradation system (ERAD) [43,44].

ATF6, once activated, translocates to the Golgi apparatus, where it is processed into a transcription factor that upregulates folding proteins such as BiP/GRP78 and PDI [45].

In all these pathways, the primary goal is protective; however, sustained activation turns this adaptive response into a signal of cell death.

### 4.2. From Protective Mechanisms to Neuronal Apoptosis

The outcome of the UPR depends on the intensity and duration of ER stress. Moderate activity induces chaperones and degradation mechanisms, whereas prolonged activation triggers apoptosis through CHOP (C/EBP homologous protein) induction and calcium release into the cytosol, which activates caspases and calpains [46].

In AD, the accumulation of β-amyloid peptide and hyperphosphorylated Tau causes ER dysfunction, leading to chronic activation of PERK and increased CHOP expression [47]. Similarly, in PD, overexpression of α-synuclein blocks protein degradation and promotes eIF2α phosphorylation, leading to dopaminergic neuronal death [48].

In ALS, mutations in ER-associated proteins such as VAPB or SIGMAR1 alter protein trafficking and calcium homeostasis, exacerbating ER stress [49]. In HD, mutant huntingtin interferes with chaperone function and sequesters ER proteins, causing a protein overload that activates the IRE1 and ATF6 pathways [50].

### 4.3. Protein Quality Control: Proteasome and Autophagy

The degradation of misfolded proteins depends on two complementary systems: the ubiquitin–proteasome system and lysosomal autophagy. The first recognizes cytosolic or ER proteins tagged with ubiquitin chains, which are then degraded by the 26S proteasome. In the second system, protein aggregates and damaged organelles are engulfed by autophagosomes and fused with lysosomes for degradation [51].

During neurodegeneration, both systems become impaired. In AD, proteasomal activity is reduced, favoring the accumulation of ubiquitinated proteins [52]. In PD, Lewy bodies contain ubiquitinated proteins, indicating proteolytic failure [53]. In ALS, inclusions of misfolded TDP-43 and SOD1 have been identified, which are resistant to proteolysis [54]. In HD, mutant huntingtin accumulates in nuclear inclusions, overwhelming proteasomal capacity [54].

Autophagy plays a critical role in eliminating aggregates and damaged mitochondria (mitophagy). In animal models, inhibition of key genes such as ATG5 or ATG7 results in spontaneous neuronal degeneration [55]. However, dysregulated autophagy activation can also be detrimental, as excessive degradation of cellular components compromises neuronal viability [56].

### 4.4. Interaction Between UPR, Mitochondria, and Oxidative Stress

ER stress and mitochondrial dysfunction form an interdependent pathological axis. The release of calcium from the ER to mitochondria through contact regions called MAMs (mitochondria-associated membranes) modulates ATP generation; however, excessive calcium flux induces mitochondrial overload and cytochrome c release. Moreover, mitochondrial reactive oxygen species amplify ER stress by oxidizing chaperones and folding-related proteins [57,58]. This feedback loop perpetuates damage and precipitates apoptosis.

### 4.5. Protein Aggregation and Prion-like Toxicity

A consequence of proteostatic failure is the formation of insoluble protein aggregates, which are not only toxic to the producing cell but can also spread between neurons through prion-like mechanisms [59]. In AD, Aβ and Tau oligomers act as seeds that induce misfolding of normal proteins [60].

In PD, misfolded α-synuclein propagates through exosomes or nanotubes [61]. In ALS, intercellular propagation of TDP-43 and SOD1 has been observed, while in HD, the mutant protein can be transmitted to neighboring neurons, contributing to the spread of damage [62].

These findings have transformed the concept of neurodegeneration from a focal pathology to a dynamic, system-wide molecular process.

Experimental models further validate these mechanisms. Tau transgenic mice exhibit chronic activation of PERK and increased CHOP expression following ER stress [63], while α-synuclein-overexpressing dopaminergic cultures demonstrate impaired proteasomal degradation and accumulation of ubiquitinated proteins [64]. In ALS models, mutation-bearing SOD1 mice show ER fragmentation and defective ER–mitochondria communication [65]. These studies provide direct cellular and animal evidence for the proteostatic impairments described.

## 5. Disruption of Calcium Homeostasis and Cellular Signaling

Calcium (Ca^2+^) is an essential second messenger in neurons, involved in neurotransmission, synaptic plasticity, gene expression, and cell survival. Ca^2+^ homeostasis requires precise control of its cytosolic concentration through a balance between influx and efflux mediated by membrane channels, pumps, and intracellular stores such as the ER and mitochondria [66]. In neurodegenerative diseases, this balance is disrupted, leading to Ca^2+^ overload, alterations in signal transduction, and activation of proteolytic and apoptotic enzymes [67].

Calcium dysregulation represents a pathogenic convergence point among oxidative stress, protein misfolding, and mitochondrial dysfunction, all of which contribute to both the early stages of neuronal injury and its propagation across synapses.

### 5.1. Calcium Channels and Transporters in Neurons

Ca^2+^ enters the neuronal cytoplasm through voltage-gated calcium channels (VGCCs), NMDA and AMPA receptors on the postsynaptic membrane, and ryanodine (RyR) or inositol triphosphate (IP3R) receptors in the ER [68,69,70]. Calcium ATPases (PMCA and SERCA) and sodium–calcium exchangers (NCX) restore basal concentrations [71].

During physiological activity, transient Ca^2+^ pulses regulate neurotransmitter release and synaptic potentiation. However, excessive or sustained activation of glutamatergic receptors—especially NMDA receptors—causes massive Ca^2+^ influx, leading to excitotoxicity, which is well described in diseases such as AD, ALS, and HD [72]. In this context, glutamate acts as an endogenous neurotoxin by overstimulating postsynaptic receptors that promote uncontrolled Ca^2+^ entry.

### 5.2. Excitotoxicity and Neuronal Vulnerability

Excitotoxicity is characterized by an increase in cytosolic Ca^2+^, which activates proteases (calpains), phospholipases, endonucleases, and neuronal nitric oxide synthase (nNOS) [73].

These events cause cytoskeletal damage, lipid peroxidation, and DNA fragmentation. In AD, the accumulation of β-amyloid peptide enhances Ca^2+^ entry through NMDA and L-type channels, sensitizing neurons to excitotoxicity [74].

In ALS, astrocytes exhibit reduced glutamate uptake due to loss of the EAAT2 transporter, increasing motor neuron exposure to toxic glutamate levels [75].

In HD, mutant huntingtin enhances the activity of NMDA and RyR receptors, exacerbating Ca^2+^ flux and accelerating neuronal degeneration [76].

### 5.3. Communication Between the ER and Mitochondria

In mitochondrial-associated membranes (MAMs), Ca^2+^ is directly transferred via ER IP_3_R channels and the mitochondrial outer membrane transporter VDAC, facilitated by the chaperone GRP75. Under physiological conditions, this communication synchronizes ATP production with synaptic energy demands. However, excessive Ca^2+^ flux to mitochondria causes mitochondrial overload, opening of the permeability transition pore (mPTP), and cytochrome c release, activating the apoptotic cascade [77,78].

In models of AD and PD, an increase in physical ER–mitochondria contact has been observed, accompanied by mitochondrial Ca^2+^ overload and loss of membrane potential [79,80].

These findings reinforce the idea that Ca^2+^ signaling disruption not only affects neuronal excitability but also bioenergetics and cell viability.

### 5.4. Calcium-Associated Proteins in Neurodegeneration

Several Ca^2+^-regulating proteins play roles in the pathogenesis of neurodegenerative diseases. Calbindin-D28k, a calcium-binding protein, is reduced in vulnerable dopaminergic neurons in PD, lowering their buffering capacity against Ca^2+^ peaks, leading to cytoskeletal degradation, and promoting Tau phosphorylation via activation of CDK5 and GSK3β [81,82].

Similarly, the Sigma-1 receptor (SIGMAR1), located at MAMs, modulates ER–mitochondria communication and contributes to neuronal survival. Mutations in the SIGMAR1 gene have been linked to familial ALS cases [83,84]. Loss of this protein disrupts calcium homeostasis, aggravating ER stress.

### 5.5. Long-Term Consequences and Relationship with Other Pathways

Chronic Ca^2+^ dysregulation not only triggers apoptosis but also impairs synaptic plasticity and memory processes. In AD, Ca^2+^ excess reduces long-term potentiation and promotes long-term depression, deteriorating neuronal connectivity [67]. In PD, Ca^2+^ oscillations in dopaminergic neurons generate metabolic vulnerability, while in HD and ALS, persistent Ca^2+^ elevations contribute to caspase activation and progressive degradation of synaptic proteins [85,86].

Ca^2+^ imbalance also intersects with other neurodegenerative pathways: it promotes ROS generation, exacerbates ER stress, impairs autophagy, and activates NF-κB, amplifying inflammation [87,88].

## 6. Neuroinflammation and Glial Reactivity

Neuroinflammation is a fundamental pathophysiological process in the progression of neurodegenerative diseases. Although under normal conditions it serves a protective function—facilitating debris clearance and promoting tissue repair—its chronic activation generates a cytotoxic environment that exacerbates neuronal damage [89]. The inflammatory response of the CNS is primarily mediated by microglia and astrocytes, whose homeostatic functions shift into reactive phenotypes in response to stimuli such as misfolded proteins, axonal injury, or synaptic dysfunction [90,91].

The balance between resolving and chronic inflammation determines whether neuronal tissue recovers or progresses toward irreversible degeneration.

### 6.1. Microglia: From Protection to Proinflammation

Microglia, derived from the myeloid lineage, act as the resident immune cells of the brain. Under physiological conditions, they maintain a ramified morphology and continuously monitor the synaptic microenvironment via receptors sensitive to damage- or pathogen-associated signals [90].

Microglia can adopt different phenotypes in response to pathological stimuli:

(a) M2 phenotype (neuroprotective): releases trophic factors (BDNF, IGF-1), enhances phagocytosis, and promotes tissue repair.
(b) M1 phenotype (proinflammatory): secretes cytokines such as IL-1β, TNF-α, and IL-6, along with nitric oxide (NO) and ROS, contributing to neuronal injury [92,93,94].

In AD and ALS, constant exposure to protein aggregates (Aβ, α-synuclein) maintains microglia in a persistent M1 state, resulting in chronic inflammation and the release of toxic mediators. In ALS, mutations in SOD1 or TDP-43 impair microglial phagocytic capacity while enhancing its inflammatory activity [95,96].

Similarly, in HD, aberrant huntingtin expression in microglia increases IL-6 and NO production, exacerbating neuronal vulnerability [97].

### 6.2. Reactive Astrocytes and Loss of Homeostasis

Astrocytes are essential for maintaining the neuronal environment: they regulate ionic balance, recycle neurotransmitters, modulate synaptogenesis, and contribute to the integrity of the blood–brain barrier. However, under conditions of neuronal injury or chronic inflammation, they can transform into reactive astrocytes characterized by overexpression of GFAP (glial fibrillary acidic protein) and morphological–functional changes [98].

A functional classification of reactive astrocytes has been proposed:

(a) A2 astrocytes: induced by ischemia, secrete neurotrophic factors, and promote repair.
(b) A1 astrocytes: induced by microglial cytokines (IL-1α, TNF-α, C1q), acquire a neurotoxic phenotype that releases proinflammatory compounds and reduces functional capacity [99].

In AD and PD, A1 astrocytes predominate, contributing to synaptic loss and inhibition of neurogenesis [100]. In ALS, the conversion of astrocytes to a toxic A1 state precedes motor neuron death, highlighting their causal role [101]. In HD, astrocytic dysfunction impairs glutamate uptake and worsens neuronal excitotoxicity [102].

### 6.3. Cytokines, Inflammasomes, and Signaling Pathways

Cytokines released by microglia and astrocytes act as key mediators of neuroinflammation. Among the most prominent are IL-1β, TNF-α, IL-6, IL-18, and TGF-β, whose elevated levels correlate with the severity of neuronal damage. These molecules activate intracellular pathways such as NF-κB, MAPK, and JAK/STAT, promoting the expression of proinflammatory and cell-death genes [103,104].

Of particular importance is the NLRP3 inflammasome, a multiprotein complex that detects danger signals (DAMPs) and activates caspase-1, which mediates the maturation of IL-1β and IL-18. In AD, inflammasome activation by Aβ oligomers induces a self-amplifying inflammation that accelerates pathology [105]. In PD, aggregated α-synuclein activates NLRP3 in microglia, promoting dopaminergic neurotoxicity [106]. In ALS and HD models, caspase-1 activation and IL-1β release are associated with motor progression and cortical atrophy [107].

Nitric oxide (NO), produced by inducible nitric oxide synthase (iNOS), also plays a dual role. At physiological concentrations, it regulates vasodilation and neurotransmission; however, in excess, it reacts with superoxide to form peroxynitrite (ONOO^−^), a highly toxic radical that oxidizes lipids and proteins, compromising synaptic integrity [108].

### 6.4. Glia–Neuron Communication and Sustained Damage

The bidirectional interaction between glia and neurons perpetuates the cycle of injury. Damaged neurons release DAMPs such as ATP, HMGB1, and DNA fragments, which activate microglial receptors like P2X7 and TLR4, promoting the release of proinflammatory cytokines. In turn, mediators released by microglia induce dysfunction in astrocytes and neurons, increasing glutamate and ROS release [109].

This pathological synergy amplifies neuroinflammation and contributes to synaptic loss—an early and common event in most neurodegenerative disorders. In advanced stages, chronic inflammation leads to disruption of the blood–brain barrier, allowing infiltration of peripheral immune cells (T lymphocytes and monocytes) that intensify the immune response [110].

Microglial activation is strongly supported by experimental data. In APP/PS1 mouse models, microglia shift toward a proinflammatory phenotype with high IL-1β and TNF-α release [111], while LPS-stimulated primary microglia exhibit enhanced NLRP3 inflammasome activity [112]. In ALS SOD1 models, microglial and astrocytic reactivity precede motor neuron death [113]. These findings demonstrate the translational relevance of glial responses discussed in this review.

In summary, neuroinflammation is not an isolated event but an integrative pathological module that interacts with oxidative stress, mitochondrial dysfunction, and proteostatic imbalance, forming a feedback network that drives the progression of neurodegeneration.

## 7. Synaptic Dysfunction and Neuronal Death

Synaptic dysfunction and neuronal death represent the final stages of the neurodegenerative process and constitute the structural basis of the cognitive, motor, and behavioral decline observed in various CNS diseases. These stages are not abrupt events but the culmination of multiple convergent pathological mechanisms—oxidative stress, inflammation, mitochondrial dysfunction, calcium dysregulation, and proteostatic collapse—that progressively compromise the functional integrity of neuronal networks [114].

In the early stages, the damage is predominantly synaptic, while in later phases, irreversible neuronal loss occurs through different forms of programmed cell death, including apoptosis, necroptosis, and ferroptosis.

### 7.1. Alterations in Synaptic Plasticity

Synaptic plasticity—the ability of synapses to strengthen or weaken their efficacy—depends on calcium regulation, neurotransmitter release, and the structural integrity of pre- and postsynaptic cytoskeletons. Under pathological conditions, the accumulation of misfolded proteins, overproduction of ROS, and prolonged glutamate exposure alter synaptic dynamics [115].

In AD, Aβ oligomers interfere with NMDA and AMPA receptor function, reducing long-term potentiation (LTP) and increasing long-term depression (LTD), processes essential for memory and learning [116]. Additionally, hyperphosphorylated Tau detaches from microtubules and redistributes to dendrites, where it disrupts synaptic function and cytoskeletal organization [117].

In PD, dopamine loss in striatal circuits disrupts excitatory/inhibitory signaling balance, impairing dopamine-dependent plasticity [118]. In ALS, glutamate reuptake impairment by astrocytes and axonal damage reduces effective transmission at neuromuscular junctions [119]. In HD, NMDA receptor dysfunction and dendritic spine loss in the striatum precede overt neurodegeneration [120].

These early synaptic alterations mark the transition from a reversible phase to an irreversible stage of neuronal injury.

### 7.2. Apoptosis: The Classical Pathway of Neuronal Death

Apoptosis is an orderly, genetically regulated form of cell death characterized by chromatin condensation, DNA fragmentation, and caspase activation. In neurodegeneration, apoptotic pathways can be triggered by intrinsic (mitochondrial) or extrinsic (death receptor-mediated) signals [121].

In the intrinsic pathway, cytochrome c release from mitochondria activates the apoptosome complex (Apaf-1, procaspase-9), initiating a cascade of effector caspases 3 and 7. This process is regulated by the Bcl-2 protein family, which includes pro-apoptotic (Bax, Bak) and anti-apoptotic (Bcl-2, Bcl-xL) factors [122]. In AD, Bax overexpression and Bcl-2 reduction correlate with disease progression [123]. In PD, loss of Parkin and PINK1 function impairs mitophagy, leading to the accumulation of damaged mitochondria that release cytochrome c [124].

The extrinsic pathway, activated by the binding of ligands such as FasL or TNF-α to their respective receptors, triggers caspase-8 activation and converges on effector caspase activation. In ALS, Fas-mediated signaling contributes to motor neuron degeneration [125]. In HD, mutant huntingtin sensitizes neurons to apoptotic stimuli through mitochondrial dysfunction and enhanced ER stress [126].

### 7.3. Necroptosis: Regulated Inflammatory Cell Death

Necroptosis is a form of programmed cell death with necrotic-like morphology but governed by specific signaling pathways. It involves activation of TNF receptors and phosphorylation of kinases RIPK1 and RIPK3, which subsequently activate MLKL (mixed lineage kinase domain-like protein). MLKL forms pores in the plasma membrane, releasing DAMPs that amplify inflammation [127].

In AD and PD models, increased phosphorylation of MLKL and activation of RIPK1 have been detected in areas of neuronal degeneration [128,129]. In ALS, necroptosis is associated with proinflammatory microglial activation and the release of toxic mediators that accelerate motor neuron loss. Pharmacological inhibition of RIPK1 has shown neuroprotective effects in animal models, suggesting therapeutic potential [130].

### 7.4. Ferroptosis: Iron- and Lipid-Dependent Cell Death

Ferroptosis is an emerging form of cell death associated with iron accumulation and lipid peroxidation. It is characterized by inhibition of glutathione peroxidase 4 (GPx4), the enzyme responsible for reducing lipid peroxides. When glutathione (GSH) is depleted or GPx4 activity decreases, oxidized lipid species accumulate and damage the cell membrane [131].

Iron plays a dual role: it is essential for mitochondrial function, yet its excess catalyzes the Fenton reaction, generating hydroxyl radicals. In AD, iron accumulates in the hippocampus and cortex, promoting Aβ oxidation and Tau phosphorylation [132]. In PD, the substantia nigra exhibits high ferric iron levels that facilitate α-synuclein aggregation [133]. In HD, impaired iron homeostasis contributes to oxidative damage and striatal neuronal loss [134].

Identifying ferroptosis as a final pathway of neuronal death opens new therapeutic perspectives based on lipophilic antioxidants (ferrostatin-1, liproxstatin-1) or iron chelators [135].

### 7.5. Interactions Among Neuronal Death Pathways

In neurodegeneration, distinct forms of cell death do not occur in isolation but coexist and interact. Oxidative stress and Ca^2+^ overload can trigger both apoptosis and necroptosis, whereas chronic inflammation and mitochondrial dysfunction favor ferroptosis. Caspases can modulate necroptosis, and ROS can amplify DAMP release, establishing a self-perpetuating cycle [136].

Understanding this interconnected network has reshaped the classical view of neuronal death as a passive event, framing it instead as a dynamic process of cellular decision-making dependent on the type of damage, metabolic context, and surrounding glial responses.

## 8. Misfolded Proteins and Their Propagation

One of the most important conceptual advances in modern neuroscience has been the recognition that misfolded proteins are not static entities confined to isolated neurons but can propagate between cells following defined anatomical patterns, in a process similar to infectious prions. This phenomenon, known as prion-like propagation, has transformed the understanding of neurodegeneration—from a focal process to a dynamic and systemic one—in which pathological proteins act as seeds that induce the conversion of native proteins into abnormal conformations [59].

The main proteins implicated in this mechanism—Tau, α-synuclein, Aβ, TDP-43, and mutant huntingtin—share the ability to self-aggregate, form fibrillar structures resistant to degradation, and transmit between neurons and glia, perpetuating the cycle of damage [137].

### 8.1. Nature of the Propagation

At the molecular level, prion-like propagation involves three fundamental stages:

(a) Nucleation: formation of a metastable intermediate nucleus competent for elongation, which does not necessarily exhibit a fully β-sheet-rich structure at this stage [138].
(b) Elongation: aggregate growth through recruitment of native proteins [139].
(c) Propagation: release of fragments or fibrils that are taken up by neighboring cells [140].

Unlike classical prions (such as PrP^Sc in spongiform encephalopathies), prion-like proteins are not infectious in an epidemiological sense, but they share a self-templating conformational mechanism. The structure of aggregates determines their ability to propagate and their degree of toxicity, generating conformational “strains” with distinct biological properties [141].

### 8.2. Propagation of Tau and β-Amyloid in Alzheimer’s Disease

In AD, the two main pathological agents—Aβ and hyperphosphorylated Tau—display distinct but complementary patterns of dissemination. Aβ accumulates extracellularly in senile plaques, whereas Tau forms intracellular aggregates known as neurofibrillary tangles. Histopathological and neuroimaging studies have shown that the spread of Tau pathology correlates more closely with clinical progression than amyloid burden [142,143,144].

Tau propagation follows a hierarchical pattern, beginning in the entorhinal cortex and spreading to the hippocampus and neocortical regions. This process appears to depend on synaptic connectivity, as interconnected neurons are more susceptible to uptake of pathological Tau released via exocytosis or extracellular vesicles [145]. Aβ, in turn, can induce Tau phosphorylation and aggregation, establishing a synergistic axis that accelerates neurodegeneration [146].

### 8.3. α-Synuclein and Its Propagation

α-Synuclein—the main component of Lewy bodies in PD and glial inclusions in multiple system atrophy—is a paradigmatic example of the prion-like model. This protein can be released into the extracellular space through exocytosis or exosomes, taken up by neighboring neurons via endocytosis or nanotubular connections, and thereby propagate pathology [147].

Braak and colleagues (2003) [148] proposed an ascending model of pathology: misfolded α-synuclein originates in the olfactory tract or enteric nervous system and spreads to the brainstem and cerebral cortex. This pattern aligns with the non-motor symptoms of PD—such as anosmia and constipation—that precede dopaminergic impairment [149].

Experimental models in rats have confirmed that injection of fibrillar α-synuclein into the striatum or substantia nigra induces endogenous Lewy body formation and progressive neurodegeneration, validating the anatomical propagation hypothesis [150].

### 8.4. TDP-43 and SOD1 in ALS

In ALS, the proteins TDP-43 and SOD1 adopt aberrant conformations that accumulate in the cytoplasm of motor neurons. Recent studies suggest that these aggregates can spread from cell to cell, contributing to the extension of degeneration from the brainstem to the spinal cord [151].

TDP-43, an RNA-binding protein, forms insoluble cytoplasmic inclusions when mislocalized from the nucleus. Its intercellular propagation may involve exosomes or direct contact mechanisms between neurons and glial cells [152]. Similarly, mutant SOD1 can act as a seed, inducing aggregation of native protein in a prion-like manner [153]. These observations explain the topographic progression of motor symptoms in ALS.

### 8.5. Mutant Huntingtin and Intercellular Dissemination

In HD, mutant huntingtin (mHTT)—bearing expanded CAG repeats—forms toxic intranuclear and cytoplasmic aggregates. Although it was historically considered a cell-autonomous pathology, mHTT is now known to transfer between neurons and astrocytes through extracellular vesicles or electrical synapses. The transfer of mHTT to glial cells not only spreads pathology but also exacerbates metabolic dysfunction in the neuronal environment [154,155].

This concept of intercellular dissemination also explains the regional progression of the disease: the most interconnected striatal circuits are the first to degenerate, reflecting a pattern of connectivity-dependent vulnerability.

### 8.6. Cellular Mechanisms of Transmission

The spread of pathological proteins can occur through several pathways:

(a) Exocytosis of oligomers or exosomes released into the extracellular space [156].
(b) Endocytosis or receptor-mediated uptake (LRP1, HSPG, PrP^C) [157].
(c) Nanotubular tunnels, allowing direct transfer of aggregates or dysfunctional mitochondria [158].
(d) Synaptic transmission, via activity-dependent release and uptake [159].

Exosomes—30–150 nm vesicles derived from multivesicular endosomes—are particularly relevant, as they can carry pathological proteins, RNA, and proinflammatory lipids. Once released, exosomes can travel short distances or even cross the blood–brain barrier, contributing to systemic spread of damage [160].

### 8.7. Pathophysiological and Therapeutic Implications

The intercellular propagation of misfolded proteins has redefined neurodegeneration as a *network disease*. This model explains the predictable anatomical progression observed in tauopathies, synucleinopathies, and TDP-43 proteinopathies, as well as the correlation between functional connectivity and damage extension [161].

From a therapeutic standpoint, these findings have inspired the development of strategies aimed at:

(a) Inhibiting the initial aggregation of pathological proteins (anti-Tau or anti-α-synuclein antibodies) [162].
(b) Blocking their release and intercellular transmission [163].
(c) Stimulating autophagy and proteolytic degradation mechanisms [164].
(d) Modulating phagocytic microglia to enhance clearance of extracellular aggregates [165].

These approaches integrate molecular neurobiology with brain connectomics, offering new perspectives for halting disease progression at the neuronal network level (Table 1).

Experimental transmission studies also support prion-like propagation. Injection of α-synuclein fibrils into the mouse striatum induces Lewy body pathology in interconnected regions [166], while Tau seeding assays demonstrate template-dependent aggregation in cultured neurons [167]. Likewise, mutant huntingtin aggregates spread among neurons in both organotypic slice cultures and Drosophila models [168]. These results reinforce the concept of intercellular dissemination of misfolded proteins.

## 9. Emerging Therapeutic Strategies and Perspectives

The multifactorial nature of neurodegeneration has revealed the impossibility of treating these diseases through a single-target (monotherapeutic) approach. Contemporary strategies aim to simultaneously intervene in multiple pathological pathways—restoring cellular homeostasis, reducing protein propagation, and attenuating neuroinflammation. Unlike traditional symptomatic therapies, new approaches seek to modify the course of the disease by integrating pharmacological, genetic, immunological, and computational perspectives [169].

This section discusses the most relevant advances in four therapeutic domains: modulation of oxidative and mitochondrial stress, restoration of proteostasis and the unfolded protein response (UPR), immunological and anti-inflammatory intervention, and novel biotechnological and artificial intelligence-based tools.

Given the multi-layered nature of neurodegeneration, emerging therapeutic strategies increasingly focus on restoring cellular homeostasis rather than targeting isolated molecular events. Traditional symptomatic treatments have shown limited disease-modifying effects, prompting the development of interventions aimed at modulating oxidative stress, stabilizing mitochondrial function, enhancing proteostasis, regulating the unfolded protein response (UPR), and attenuating chronic neuroinflammation [170]. Recent advances also include the refinement of immunotherapies directed against misfolded proteins, gene-editing tools capable of correcting pathogenic mutations, and cellular reprogramming techniques that seek to replace lost neuronal populations [171]. Furthermore, computational approaches and artificial intelligence have accelerated drug repurposing and candidate identification [172,173]. Together, these strategies reflect a paradigm shift toward multi-target and systems-based interventions, opening new avenues for modifying the course of neurodegenerative diseases.

### 9.1. Modulation of Oxidative Stress and Mitochondrial Function

Since oxidative stress and mitochondrial dysfunction are early events in neurodegeneration, one of the most explored strategies involves strengthening antioxidant systems and improving neuronal bioenergetics.

Direct antioxidants such as coenzyme Q10, alpha-lipoic acid, and N-acetylcysteine have shown limited clinical benefits due to their poor penetration into the CNS. Conversely, Nrf2 modulators like dimethyl fumarate and sulforaphane have demonstrated endogenous activation of antioxidant enzymes (GPx, SOD, HO-1), exhibiting neuroprotective effects in AD and PD models [174].

Another promising approach is to enhance mitochondrial biogenesis through activation of the coactivator PGC-1α or sirtuins (SIRT1), which regulate genes involved in energy metabolism and mitochondrial DNA repair. Compounds such as resveratrol and nicotinamide riboside have produced encouraging preclinical results [175].

### 9.2. Restoration of Proteostasis and Modulation of the UPR

Restoring proteostasis remains a key therapeutic goal, as the accumulation of misfolded proteins is a central feature of neurodegeneration. Pharmacological chaperones (e.g., arimoclomol and celastrol) improve protein folding and reduce endoplasmic reticulum stress. In ALS, arimoclomol enhanced HSP70 expression, although clinical outcomes were modest [176,177].

UPR modulators offer a promising avenue to prevent the transition from an adaptive to an apoptotic response. The PERK inhibitor GSK2606414 prevented synaptic loss in tauopathy models, though with systemic side effects [178]. Alternatively, the small molecule ISRIB (Integrated Stress Response Inhibitor) restores protein translation without fully suppressing the UPR, showing positive preclinical outcomes in AD and PD models [179].

Additionally, stimulation of autophagy via rapamycin or AMPK activators enhances the clearance of aggregated proteins and damaged mitochondria, contributing to neuroprotection [180].

### 9.3. Immunological and Anti-Inflammatory Intervention

Chronic neuroinflammation perpetuates neuronal degeneration and has become a major therapeutic target aimed at modulating glial responses.

Traditional anti-inflammatories (such as COX-2 inhibitors) have shown limited efficacy in advanced stages, likely due to the loss of tissue plasticity. Consequently, current efforts focus on reprogramming microglia toward a reparative M2 phenotype via TREM2 modulators, fractalkine (CX3CL1), or PPARγ agonists [181].

Immunotherapies targeting pathological proteins are another rapidly developing approach. In AD, the antibodies aducanumab [182] and lecanemab [183] have reduced amyloid burden, though with partial clinical efficacy and adverse event risks. In PD, anti-α-synuclein antibodies (prasinezumab) are being tested to block prion-like propagation [184]. In tauopathies, anti-Tau antibodies (gosuranemab, semorinemab) are under clinical evaluation with variable outcomes [185,186].

The development of nanoparticles targeted to the inflammatory microenvironment, capable of controlled drug release, represents a next-generation approach with potential for high specificity and reduced systemic toxicity [187].

### 9.4. Gene Therapy, Editing, and Cellular Reprogramming

Gene therapy has emerged as a powerful tool to correct causal mutations and restore neuronal functions. Delivery of functional genes via adeno-associated viral (AAV) vectors has been tested in PD (AAV-GAD in the subthalamic nucleus) and in monogenic disorders such as HD (AAV-shRNA targeting mutant HTT) [188,189].

At the epigenetic level, histone deacetylase (HDAC) inhibitors and DNA methylation modulators have demonstrated the ability to reverse transcriptional alterations in AD and ALS models [190,191]. Likewise, in vivo reprogramming of astrocytes into functional neurons by expressing factors such as NeuroD1 or Ascl1 represents an innovative strategy for restoring damaged circuits [192,193,194] (Table 2).

## 10. Discussion

Neurodegeneration represents a convergent endpoint of diverse molecular insults that disrupt the finely tuned balance of neuronal homeostasis. Despite the distinct etiologies of AD, PD, ALS, and HD, the evidence synthesized in this work supports a shared pathological framework governed by oxidative stress, mitochondrial dysfunction, proteostatic collapse, excitotoxicity, and chronic inflammation. These overlapping mechanisms form a multidimensional network rather than independent cascades, explaining the progressive and system-wide nature of neuronal loss [195].

### 10.1. Interdependence of Cellular Stress Pathways

Oxidative stress and mitochondrial impairment emerge as early and self-amplifying events that compromise energy metabolism and redox balance. Reactive oxygen species not only damage lipids and DNA but also oxidize chaperones and endoplasmic reticulum (ER) proteins, aggravating unfolded protein response (UPR) activation. In turn, chronic ER stress disrupts calcium handling and enhances mitochondrial permeability, creating a positive feedback loop that links bioenergetic failure with proteostatic dysfunction. The interplay between these organelles—mediated through mitochondrial-associated membranes (MAMs)—is now recognized as a key axis of neuronal vulnerability [196,197].

### 10.2. Glial Reactivity and the Inflammatory Amplification Cycle

The transition from an adaptive to a chronic inflammatory state constitutes a turning point in neurodegeneration. Activated microglia and reactive astrocytes shift from neuroprotective to neurotoxic phenotypes, releasing cytokines, nitric oxide, and reactive species that further destabilize neuronal circuits. The persistent activation of inflammasomes such as NLRP3 integrates molecular stress with immune responses, bridging protein misfolding and inflammation. This glial–neuronal crosstalk amplifies neurotoxicity, eroding synaptic integrity and perpetuating the degenerative cycle across neural networks [198].

### 10.3. Synaptic Vulnerability and Network Disintegration

Synaptic dysfunction precedes overt neuronal loss, underscoring the notion that neurodegeneration begins as a failure of communication rather than a massive cell death phenomenon. Dysregulation of calcium dynamics and glutamate signaling drives excitotoxicity, which progressively disconnects neural circuits. In parallel, the prion-like propagation of misfolded proteins such as Tau, α-synuclein, TDP-43, and mutant huntingtin reveals that neurodegeneration spreads along anatomical and functional connectivity maps, transforming localized molecular stress into global network pathology [199].

An additional element that contributes to the system-level progression of neurodegeneration is the molecular crosstalk among distinct pathological amyloids. Increasing evidence shows that Aβ, Tau, α-synuclein, TDP-43, and mutant huntingtin can influence each other’s aggregation dynamics through heterotypic seeding, cooperative fibrillization, or shared proteostatic and inflammatory pathways [200]. For example, Aβ can accelerate Tau phosphorylation and aggregation, while α-synuclein seeds can promote Tau fibrillization in vitro and in vivo [146]. Similarly, TDP-43 and α-synuclein aggregates interact in ALS and PD, contributing to overlapping clinical phenotypes. These hetero-amyloid interactions suggest that neurodegenerative diseases are not isolated proteinopathies but interconnected network disorders in which misfolded proteins amplify each other’s toxicity and spread [201].

### 10.4. Integration of Cell Death Mechanisms

Apoptosis, necroptosis, and ferroptosis represent distinct yet interconnected execution pathways of neuronal death. Mitochondrial dysfunction and oxidative imbalance provide a common ground for their activation, while inflammatory mediators modulate the decision between regulated and necrotic death. The convergence of these pathways emphasizes that neuronal demise is not a binary event but a dynamic continuum dictated by metabolic stress, glial signaling, and genomic instability [202].

### 10.5. Toward Multi-Target and Systems-Based Therapies

The therapeutic section of this work highlights that no single pharmacological agent can reverse the multifactorial complexity of neurodegeneration. Instead, integrative interventions—combining antioxidants, proteostasis modulators, anti-inflammatory agents, gene therapies, and computationally optimized drug repurposing—represent the most rational approach. Systems biology and artificial intelligence offer powerful frameworks for mapping molecular interactions and predicting synergistic therapeutic combinations. Future strategies should prioritize early intervention, glial modulation, and personalized multi-drug regimens adapted to the specific stage and molecular profile of each disease [203,204].

From a translational perspective, the integrative framework presented in this review has several practical applications. Understanding how oxidative stress, mitochondrial dysfunction, proteostasis collapse, neuroinflammation, and prion-like propagation interact offers a conceptual basis for designing multi-target therapies rather than single-pathway interventions. This systems-level approach may guide the development of biomarker panels that capture the dynamic progression of neurodegeneration, improve patient stratification in clinical trials, and inform the timing of therapeutic intervention—particularly in preclinical or prodromal stages. Moreover, mapping convergent mechanisms across AD, PD, ALS, and HD facilitates drug repurposing by identifying shared molecular nodes that can be targeted across disorders. These applications underscore the clinical relevance of integrating mechanistic knowledge into therapeutic design and highlight the importance of interdisciplinary approaches that combine molecular biology, neuroimaging, computational modeling, and immunological profiling.

Consistent with the introductory nature of this review, the aim was not to provide exhaustive coverage of every molecular pathway but to integrate the most convergent and well-established mechanisms into a unified conceptual model.

### 10.6. Conceptual Implications

This integrative view reframes neurodegeneration not as a linear cascade but as a self-propagating systems disorder involving neurons, glia, and their metabolic environment. Understanding its emergent properties—feedback loops, crosstalk among organelles, and network-level propagation—will be crucial for developing disease-modifying therapies. Ultimately, restoring the resilience of neuronal systems requires shifting from isolated molecular targets to the orchestration of cellular homeostasis across multiple biological scales.

## 11. Conclusions

As an introductory overview, this review synthesizes the major convergent mechanisms rather than examining each pathway in exhaustive depth.

The prospect of achieving a complete cure for neurodegenerative diseases remains uncertain, as no single drug can inhibit all stages of the neurodegenerative process. Treatment should therefore be tailored to the stage of the disease, and in advanced phases, a comprehensive therapeutic approach targeting multiple mechanisms simultaneously will be required.

At present, prevention remains our most effective strategy—living as healthily as possible and avoiding exposure to factors that disrupt neuronal homeostasis and initiate the still irreversible neurodegenerative cascade.

## Figures and Tables

**Table 1:** Stages of the Neurodegenerative Process and Main Cellular Events.

Stage	Main Cellular and Molecular Events
1. Molecular Initiation (Triggering Phase)	Genetic mutations, oxidative stress, viral or toxic exposure, or aging initiate early molecular damage. Mitochondrial DNA becomes unstable, proteins misfold, and endoplasmic reticulum (ER) stress pathways are activated.
2. Proteostasis Dysfunction	Impaired folding and clearance of proteins lead to toxic aggregates such as amyloid-β plaques, Tau tangles, α-synuclein Lewy bodies, or huntingtin inclusions. Ubiquitin–proteasome and autophagy-lysosome systems fail to maintain protein homeostasis.
3. Oxidative Stress and Mitochondrial Dysfunction	Excessive production of reactive oxygen and nitrogen species (ROS/RNS) damages lipids, proteins, and DNA. Mitochondrial membrane potential decreases, leading to energy failure, calcium dysregulation, and apoptotic signaling.
4. Chronic Neuroinflammation	Microglia and astrocytes become chronically activated, releasing proinflammatory cytokines (IL-1β, TNF-α, IL-6) and reactive species. The inflammatory cycle amplifies neuronal injury and disrupts glial homeostatic functions.
5. Synaptic Dysfunction	Neurotransmission becomes impaired: cholinergic loss (Alzheimer’s), dopaminergic depletion (Parkinson’s), and glutamatergic excitotoxicity (ALS). Reduced dendritic spines and synaptic plasticity cause network disconnection and cognitive decline.
6. Programmed Neuronal Death	Neurons undergo apoptosis, necroptosis, or ferroptosis. Caspase activation, mitochondrial release of cytochrome c, and iron accumulation lead to irreversible neuronal loss in vulnerable regions (e.g., hippocampus, substantia nigra).
7. Prion-like Propagation and Feedback Loop	Misfolded proteins spread between cells via exosomes or direct contact, seeding further aggregation. The cycle of oxidative stress, inflammation, and proteotoxicity reinforces neurodegeneration and clinical progression.

**Table 2:** Emerging Therapeutic Strategies and Perspectives in Neurodegeneration.

Therapeutic Axis	Strategy/Mechanism	Molecules/Interventions	Model or Disease	Main Findings	Reference
Modulation of Oxidative Stress and Mitochondrial Function	Activation of Nrf2 and antioxidant enzymes	Dimethyl fumarate, sulforaphane	Alzheimer’s, Parkinson’s	Induces GPx, SOD, and HO-1 expression; neuroprotective effects.	[174]
	Enhancement of mitochondrial biogenesis	Resveratrol, nicotinamide riboside	Preclinical models	Activation of PGC-1α and SIRT1; improved neuronal metabolism.	[175]
Restoration of Proteostasis and UPR Modulation	Induction of molecular chaperones	Arimoclomol, celastrol	ALS	Upregulation of HSP70; modest clinical outcomes.	[176]
	Selective inhibition of PERK	GSK2606414	Tauopathies	Prevented synaptic loss; systemic toxicity observed.	[178]
	Restoration of protein translation	ISRIB (Integrated Stress Response Inhibitor)	Alzheimer’s, Parkinson’s	Restores protein synthesis without full UPR suppression; preclinical neuroprotection.	[179]
	Stimulation of autophagy	Rapamycin, AMPK activators	Cellular and animal models	Promotes clearance of protein aggregates and damaged mitochondria.	[180]
Immunological and Anti-Inflammatory Intervention	Microglial reprogramming toward M2 phenotype	TREM2 modulators, fractalkine (CX3CL1), PPARγ agonists	Alzheimer’s, Parkinson’s	Induces anti-inflammatory and reparative microglial phenotype.	[181]
	Anti-β-amyloid immunotherapy	Aducanumab, lecanemab	Alzheimer’s	Reduces amyloid burden; partial clinical efficacy with side effects.	[182,183]
	Anti-α-synuclein immunotherapy	Prasinezumab	Parkinson’s	Inhibits prion-like propagation of α-synuclein aggregates.	[184]
	Anti-Tau immunotherapy	Gosuranemab, semorinemab	Tauopathies	Variable outcomes; clinical trials ongoing.	[185,186]
	Targeted nanoparticles for controlled drug delivery	Nanoparticle-based drug release systems	Neurodegenerative models	Controlled release with high specificity and reduced systemic toxicity.	[187]
Gene Therapy, Editing, and Cellular Reprogramming	AAV-based gene delivery	AAV-GAD (subthalamic nucleus, PD), AAV-shRNA-HTT (Huntington’s)	Parkinson’s, Huntington’s	Restored neuronal function and reduced mutant gene expression.	[188,189]
	Epigenetic modulation	HDAC inhibitors, DNA methylation modulators	Alzheimer’s, ALS	Reversal of transcriptional alterations in neurodegenerative models.	[190,191]
	In vivo cellular reprogramming	NeuroD1, Ascl1 expression	Murine models	Conversion of astrocytes into functional neurons; restoration of damaged circuits.	[192,193,194]

## Data Availability

No new data were created or analyzed in this study. Data sharing is not applicable to this article.

## Abbreviations

The following abbreviations are used in this manuscript:

A1	Reactive astrocyte subtype (neurotoxic)
A2	Reactive astrocyte subtype (neuroprotective)
AAV	Adeno-associated virus
AAV-GAD	AAV carrying glutamic acid decarboxylase gene
AAV-shRNA-HTT	AAV delivering short hairpin RNA against mutant huntingtin
Aβ	Amyloid-beta peptide
AD	Alzheimer’s disease
ALS	Amyotrophic lateral sclerosis
AMPA	α-amino-3-hydroxy-5-methyl-4-isoxazolepropionic acid receptor
AMPK	AMP-activated protein kinase
Apaf-1	Apoptotic protease-activating factor 1
ATF4	Activating transcription factor 4
ATF6	Activating transcription factor 6
ATP	Adenosine triphosphate
BDNF	Brain-derived neurotrophic factor
BiP/GRP78	Binding immunoglobulin protein/Glucose-regulated protein 78
Bcl-2	B-cell lymphoma 2 (anti-apoptotic protein)
Ca^2+^	Calcium ion
CAT	Catalase
CHOP	C/EBP homologous protein
CNS	Central nervous system
COX-2	Cyclooxygenase-2
CX3CL1	Fractalkine (chemokine ligand 1)
DAMPs	Damage-associated molecular patterns
DNA	Deoxyribonucleic acid
ΔΨm	Mitochondrial membrane potential
EAAT2	Excitatory amino acid transporter 2
ER	Endoplasmic reticulum
ERAD	Endoplasmic reticulum-associated degradation
FasL	Fas ligand
GFAP	Glial fibrillary acidic protein
GPx	Glutathione peroxidase
GPx4	Glutathione peroxidase 4
GRP75	Glucose-regulated protein 75 (mitochondrial chaperone)
GSH	Reduced glutathione
GSK3β	Glycogen synthase kinase 3 beta
HD	Huntington’s disease
HDAC	Histone deacetylase
HMGB1	High-mobility group box 1
HO-1	Heme oxygenase 1
HSP70	Heat shock protein 70
HTT	Huntingtin gene
IGF-1	Insulin-like growth factor 1
IL-1β	Interleukin-1 beta
IL-6	Interleukin-6
IL-18	Interleukin-18
iNOS	Inducible nitric oxide synthase
IP3R	Inositol 1,4,5-trisphosphate receptor
IRE1α	Inositol-requiring enzyme 1 alpha
ISRIB	Integrated stress response inhibitor
JAK/STAT	Janus kinase/Signal transducer and activator of transcription
LTP	Long-term potentiation
LTD	Long-term depression
MAPK	Mitogen-activated protein kinase
M1	Proinflammatory microglial phenotype
M2	Anti-inflammatory (reparative) microglial phenotype
MAMs	Mitochondria-associated membranes
mHTT	Mutant huntingtin
MLKL	Mixed lineage kinase domain-like protein
mPTP	Mitochondrial permeability transition pore
NF-κB	Nuclear factor kappa-light-chain-enhancer of activated B cells
NLRP3	NOD-like receptor family pyrin domain-containing 3
NMDA	N-methyl-D-aspartate receptor
NO	Nitric oxide
Nrf2	Nuclear factor erythroid 2-related factor 2
O_2_^−^	Superoxide anion
ONOO^−^	Peroxynitrite
PD	Parkinson’s disease
PDI	Protein disulfide isomerase
PGC-1α	Peroxisome proliferator-activated receptor gamma coactivator 1-alpha
PMCA	Plasma membrane calcium ATPase
PPARγ	Peroxisome proliferator-activated receptor gamma
PrP^c^	Cellular prion protein
RIPK1	Receptor-interacting protein kinase 1
RIPK3	Receptor-interacting protein kinase 3
RNA	Ribonucleic acid
RNS	Reactive nitrogen species
ROS	Reactive oxygen species
RyR	Ryanodine receptor
SIRT1	Sirtuin 1
SIGMAR1	Sigma-1 receptor
SOD	Superoxide dismutase
SOD1	Superoxide dismutase 1
TDP-43	TAR DNA-binding protein 43
TGF-β	Transforming growth factor beta
TNF-α	Tumor necrosis factor alpha
TREM2	Triggering receptor expressed on myeloid cells 2
UPR	Unfolded protein response
VDAC	Voltage-dependent anion channel
VGCCs	Voltage-gated calcium channels
